# Antigen and Cell-Based Assays for the Detection of Non-HLA Antibodies

**DOI:** 10.3389/fimmu.2022.864671

**Published:** 2022-05-06

**Authors:** Rosa G. M. Lammerts, Dania Altulea, Bouke G. Hepkema, Jan-Stephan Sanders, Jacob van den Born, Stefan P. Berger

**Affiliations:** ^1^ Transplantation Immunology, Department of Laboratory Medicine, University Medical Center Groningen, University of Groningen, Groningen, Netherlands; ^2^ Division of Nephrology, Department of Internal Medicine, University Medical Center Groningen, University of Groningen, Groningen, Netherlands

**Keywords:** non-HLA, endothelial crossmatching assays, solid-organ transplantation, solid-phase detection assays, antibody-mediated allograft rejection

## Abstract

To date, human leukocyte antigens (HLA) have been the major focus in the approach to acute and chronic antibody-mediated rejection (AMBR) in solid-organ transplantation. However, evidence from the clinic and published studies has shown that non-HLA antibodies, particularly anti-endothelial cell antibodies (AECAs), are found either in the context of AMBR or synergistically in the presence of donor-specific anti-HLA antibodies (DSA). Numerous studies have explored the influence of AECAs on clinical outcomes, yet the determination of the exact clinical relevance of non-HLA antibodies in organ transplantation is not fully established. This is due to highly heterogeneous study designs including differences in testing methods and outcome measures. Efforts to develop reliable and sensitive diagnostic non-HLA antibody tests are continuously made. This is essential considering the technical difficulties of non-HLA antibody assays and the large variation in reported incidences of antibodies. In addition, it is important to take donor specificity into account in order to draw clinically relevant conclusions from non-HLA antibody assays. Here, we provide an overview of non-HLA solid-phase and cell-based crossmatch assays for use in solid-organ transplantation that are currently available, either in a research setting or commercially.

## Introduction

**Figure 1:**
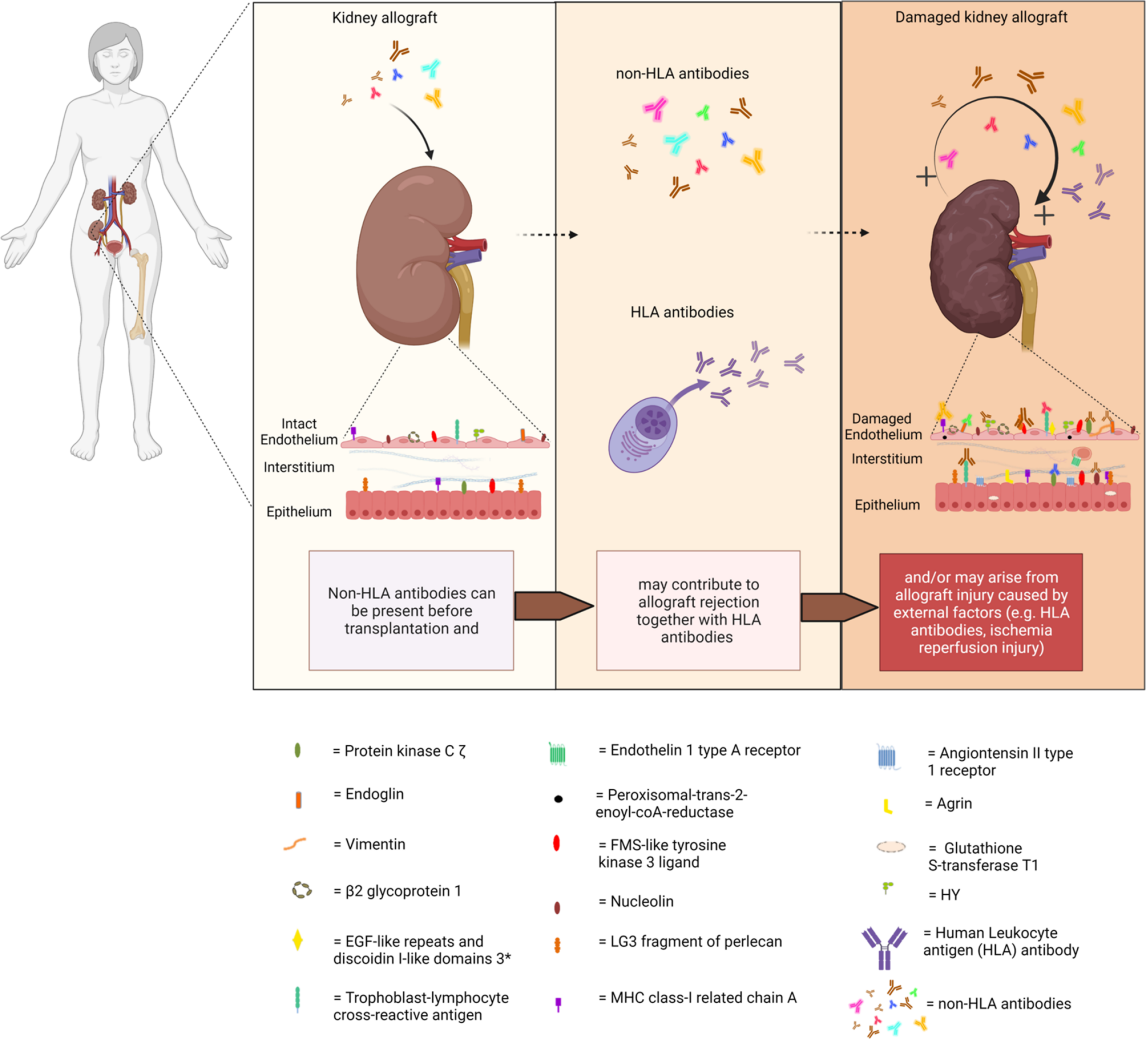
Non-HLA immunization can both contribute to and arise from allograft injury. (Non-HLA antibodies depicted in the illustration are examples, and no scientific evidence exists that it is this specific antibody that is present at this time point). Created with biorender.com.

Acute and chronic antibody-mediated rejection (ABMR) are highlighted in studies published in the last decade as an important contributor to organ allograft loss and the lack of long-term survival improvements for transplanted organs. In order to extend the outcomes of the transplanted allografts, a better understanding of the mechanisms of early and late ABMR and the development of protocols to combat and control these processes is needed (1–4). The interest in the role of donor-specific antibodies (DSA) with specificity for antigens other than human leukocyte antigen (HLA) in the contribution of the process of allograft rejection is growing (5). This has led to the identification of possible non-HLA target antigens and studies into the mechanisms of injury (6–14). However, the understanding of the effect and cause of non-HLA antibodies and the clinical importance of pre-transplant detection remains incomplete, given the fact that non-HLA immunization can both contribute to and arise from allograft injury (**Figure 1**) (15–20). Although several non-HLA antibody specificities have been identified using laboratory-developed assays, large cohort studies into the role of non-HLA antibodies have used commercially available assays (8, 21, 22). The most reported non-HLA antibodies are directed against angiotensin II type 1 receptor (AT_1_R), MHC class I chain-related antigen A (MICA), tubulin, vimentin, endothelin receptors, collagens, and anti-endothelial cell antibodies (AECAs), using either commercially available or laboratory-developed assays (6, 7, 23). However, despite a large number of published assays, screening for the presence of non-HLA antibodies has still not entered routine clinical practice in transplant medicine. Endothelial crossmatching assays have been used to detect non-HLA antibodies with flow cytometry (e.g., XM-ONE) (21, 24–27). In addition, enzyme-linked immunosorbent assay (ELISA), high-density protein arrays, indirect immunofluorescence, and serological analysis of recombinant cDNA expression libraries (SEREX) have also been described (28–30). Efforts to develop reliable and sensitive diagnostic non-HLA antibody tests capable of detecting new non-HLA antibodies are continuously made. This is essential considering the technical difficulties of non-HLA antibody assays and the large variation in reported incidences of antibodies. In addition, it is important to take donor specificity into account in order to draw conclusions from non-HLA antibody assays that have patient-related consequences.

In this review, we surveyed the current literature and provided an overview of the recently published non-HLA antibody detection and cell-based crossmatch assays developed for use in solid-organ transplantation, either in a research setting or commercially. The articles reviewed in this report were selected based on two criteria: 1) whether the aim of the study was to test a new technology for the detection of non-HLA antibodies and 2) whether the study also included a correlation between the identified non-HLA antibodies and rejection episodes (**Tables 1**–**5**).

**Table 1a:** XM-ONE studies in kidney transplantation.

Reference		Organ		Overall conclusion		Sample details^a^	Type of assay^a^		Key limitations	
Breimer et al., 2009 (21)		Kidney		Patients with pre-transplant sera positive for AECAs had a higher risk for rejection or impaired kidney function post-transplant		Pre-transplant serum samples of 147 patients were screened for AECAs	The XM-ONE assay was used to screen for AECAs. EPC-reactive IgG and IgM were detected by flow cytometry		XM-ONE uses EPCs as target cells which lack EC markers such as CD31 and CD34; no target antigens were identified	
Soyöz et al., 2020 (26)		Kidney		AECAs were not detected in the serum of all patients including the three patients who experienced biopsy-confirmed rejection		Post-transplant serum samples from 13 living donor KTRs were screened for AECAs	The XM-ONE assay was used to screen for AECAs. EPC-reactive IgG and IgM were detected by flow cytometry		The kidney donors in this study were first-degree relatives which might have improved the compliance to AECAs leading to the negative XM-ONE results	
Zitzner et al., 2013 (27)		Kidney		No association was found between the XM-ONE result and the biopsy-proven rejection or vasculopathy at 1-year post-transplant		Pre-transplant serum samples from 150 living donor KTRs were tested for AECAs	The XM-ONE assay was used to screen for AECAs. EPC-reactive IgG and IgM were detected by flow cytometry		A different immunosuppressive protocol (alemtuzumab induction) was used in comparison to other studies	
Yu et al., 2020 (31)		Kidney		The presence of AT_1_R-Abs and AECAs may contribute independently to a worse post-transplant outcome in low-risk, living donor KTRs		Levels of AT_1_R-Abs and AECAs were determined in 94 pre-transplant and 29 post-transplant serum samples in living donor KTRs with biopsy-proven rejection	AT_1_R-Abs levels were assessed with AT_1_R ELISA. The presence of AECAs was detected with XM-ONE assay		In most of the patients (65/94), only pre-transplant sera were tested for non-HLA Abs; therefore, the post-transplant impact of AT_1_R-Abs and AECAs was not reported	
Philogene et al., 2017 (12)		Kidney		The presence of and AT_1_R-Abs may contribute to the microvascular injury observed in ABMR especially in the presence of HLA-DSA		Post-transplant AT_1_R-Abs levels were measured in 70 KTRs, and AECA levels were measured in 35 KTRs who had low to negative HLA-DSA	Commercial ELISA was used to measure AT_1_R-Abs, and an XM-ONE assay was used for the AECAs		Pre-transplant serum samples were unavailable for testing; the study included hypertensive patients who underwent ARB treatment at the time of graft dysfunction	

ABMR, Antibody-mediated rejection; AECAs, anti-endothelial cell antibodies; ARB, angiotensin receptor blocker; AT1R, angiotensin type 1 receptor; EPCs, endothelial precursor cells; KTR, kidney transplant recipient.

a Sample details as well as the type of assay pertain only to the non-HLA detection method described in the article and not the entire methodology section.

**Table 1b:** Non-HLA antibody detection studies in kidney transplantation.

Reference	Organ	Overall conclusion	Sample details^a^	Type of assay^a^	Key limitations
Pontes et al., 2001 (32)	Kidney	Cultured ECs can be used to detect non-HLA Abs with IIF	A single serum sample obtained from a KTR after two rejected grafts and undetectable HLA at baseline	ECXM assay using HUVECs as the target cells. EC antigens were visualized using IIF with mouse anti-human Ig-TRITC	One post-transplant serum sample from a single patient; non-donor-derived ECs for ECXM assay; IIF has lower sensitivity
Ming et al., 2015 (33)	Kidney	MICA-DSAs in the serum were cytotoxic to ECs expressing MICA-G1 group antigens	A single serum sample positive for MICA-DSAs obtained from a KTR after a previously rejected graft with no HLA mismatch	ECXM assay using HUVECs as the target cells. Mouse anti-human IgG was used to visualize the results by flow cytometry	One post-transplant serum sample from a single patient; non-donor-derived ECs for ECXM assay; flow cytometry assays suffer from gating bias
Crespo et al., 2021 (34)	Kidney	The combination of pre-transplant HLA-DSA and AT_1_R-Abs was strongly associated with the histology of ABMR, and the post-transplant combination did not. Neither pre- nor post-transplant MICA-Abs, ETAR-Abs, or ECXM correlated with ABMR histology, with or without HLA-DSA	19 KTRs with normal histology, 52 KTRs with ABMR histology, and 47 KTRs with IFTA were screened for anti-MICA-Abs, anti-AT_1_R-Abs, and anti-ETAR-Abs, and an ECXM was performed in pre- and post-transplant sera	MICA-Abs were detected using commercial Luminex technology. Sandwich ELISAs were used to detect AT_1_R- and ETAR-Abs. ECXM assay using primary aortic endothelial cells as the target cells. EC-reactive IgG was detected by flow cytometry	Broad heterogeneity in the inclusion timing and clinical course of the patients. ECXM was not performed with renal ECs. Purification of primary EC isolation and gating strategy is not described resulting in a possible contamination with other cell types
Pearl et al., 2020 (11)	Kidney	The presence of ETAR-Abs was significantly associated with AT_1_R-Abs. These Abs were found to not be associated with ABMR or HLA-DSA development; however, they were significantly associated with microvascular injury, elevated levels of IL-8, and impaired renal function	The relationship between ETAR-Abs and AT_1_R-Abs was investigated with regard to biopsy findings, pro-inflammatory cytokine production, and HLA-DSA in a cohort of 67 pediatric KTRs post-transplant	Commercial ELISAs were used to measure AT_1_R-Abs and ETAR-Abs in the post-transplant serum samples. A custom magnetic bead kit was used to measure the cytokine levels and visualized with Luminex	Pre-transplant serum samples were not assessed; no ECXMs were performed to assess for other potential AECAs; the use of ATG increased the risk of AT_1_R-Abs development
Sorohan et al., 2021 (35)	Kidney	No relationship was found between post-transplant AT_1_R-Abs and biopsy-proven rejection	Pre- and post-transplant serum samples from 56 KTRs were screened for AT_1_R-Abs	AT_1_R-Abs were measured using a commercial quantitative ELISA	Lack of HLA-DSA in the study population made it difficult to assess the relationship between AT_1_R-Abs and HLA-DSA; biopsies were only taken by indication leading to a lack of cases with biopsy-proven rejection
Reindl-Schwaighofer et al., 2019 (36)	Kidney	Genetic mismatches of non-HLA haplotypes coding for transmembrane or secreted proteins were associated with an increased risk of functional graft loss	25 KTRs with biopsy-proven rejection were screened for the presence of non-HLA mismatches	Genetic mismatches between 477 pairs of deceased donors and first kidney transplant recipients were measured using a genome-wide analysis	The implementation of this technique as a bulk lab screening method is difficult as it is expensive and time-consuming to conduct a genome-wide analysis on a routine basis
Jackson et al., 2015 (37)	Kidney	ProtoArray identified four targets, namely, endoglin, FLT3, EDIL3, and intercellular adhesion molecule 4 which, upon further assessment *in vitro*, were associated with HLA-DSA sensitization, ABMR, and early transplant glomerulopathy	Sera from 10 KTRs from a discovery cohort experiencing ABMR in the absence of HLA-DSA were used to construct the ProtoArray. Additional sera from 150 KTRs were used to validate the ProtoArray results	ProtoArray technology was used to identify antigenic targets for AECAs. The presence of Abs against the detected targets was investigated in pre- and post-transplant serum samples with ELISAs	Many of the patients experiencing rejection post-transplant were also positive for HLA-DSA as indicated by biopsy; therefore, the association of ABMR to AECAs in these patients was confounded
Li et al., 2009 (38)	Kidney	An increase was detected in the signal for *de-novo* Abs by an average of 61% in all patients in the post-transplant serum compared with the pre-transplant. MICA-Abs were detected in 72% of the patients post-transplant	Pre- and post-transplant serum samples from 18 pediatric KTRs were examined for non-HLA antibody response	ProtoArray technology was used to identify antigenic targets for AECAs. The preferential expression of a particular antibody in the kidney tissue was investigated in 7 microdissected kidney compartments	No correlation between the development of the MICA-Abs and the risk of rejection in the recipients was tested
Clotet-Freixas et al., 2021 (10)	Kidney	Autoantibodies against Ro/SS-A (52 kDa), CENP-B, and La/SS-B were significantly elevated in KTRs with ABMR and mixed rejection compared to ACR	Serum samples from 80 KTRs were diagnosed with ABMR, ACR, mixed rejection, or acute tubular necrosis analyzed for non-HLA targets	Protein microarray platform against 134 IgG and IgM non-HLA targets was used	The study did not report the impact of these autoantibodies in patients with no HLA-DSA
Sanchez-Zapardiel et al., 2016 (9)	Kidney	Preformed MICA-Abs were able to fix and activate the complement system, therefore mediating cell death. Patients with MICA-Abs along with HLA-DSA had the worst outcomes	Serum samples from 52 KTRs were tested for MICA-Abs and C1q binding	Both MICA-Abs and C1q binding were analyzed with a Luminex platform	The study did not define whether these anti-MICA antibodies were donor specific
Kamburova et al., 2018–2019 (8, 39)	Kidney	Antibodies against ARHGDIB were more clinically relevant in deceased donor KTRs compared to living donor KTRs	Pre-transplant serum samples of 4,770 KTRs were screened for 14 non-HLA antibodies	A Luminex assay with 31 different microspheres consisting of various proteins was constructed to screen for 14 non-HLA antibodies	The multiplex assay did not investigate the presence of commonly described non-HLA targets such as AT_1_R and ETAR
Lamarthée et al., 2021 (40)	Kidney	Non-HLA Abs were increased in patients who underwent a previous kidney transplantation	Pre-transplant serum samples from an unselected cohort of 389 KTRs	Non-HLA Ab detection immunoassay (NHADIA) using CRISPR/Cas9 deleted B2M and CITTA using CiGEnCs	ECs from a single donor and from a single vascular structure. The study did not measure solid-phase non-HLA Abs

ABMR, antibody-mediated rejection; ACR, acute cellular rejection; AECAs, anti-endothelial cell antibodies; ARHGDIB, rho GDP dissociation inhibitor beta; AT_1_R, angiotensin type 1 receptor; ATG, anti-thymocyte globulin; CiGEnCs, conditionally immortalized glomerular endothelial cells; ECs, endothelial cells; ECXM, endothelial cells crossmatching; ETAR, endothelin type A receptor; IFTA, interstitial fibrosis and tubular atrophy; Ig-TRITC, immunoglobulin-tetramethylrhodamine; IIF, indirect immunofluorescence; IL-8, interleukin 8; KTR, kidney transplant recipient; MICA-DSA, major histocompatibility complex class I chain-related A donor-specific antibody; non-HLA Abs, non-human leukocyte antigen antibodies.

a Sample details as well as the type of assay pertain only to the non-HLA detection method described in the article and not the entire methodology section.

**Table 2:** Non-HLA antibody detection studies in lung transplantation.

Reference	Organ	Overall conclusion	Sample details^a^	Type of assay^a^	Key limitations
Margo et al., 2002 (28)	Lung	IIF revealed an increase in the granular nuclear and cytoplasmic staining pattern indicative of positive reactivity to ECs in 18 of 19 patients	Post-transplant sera from 19 LTRs were screened for AECAs	An ECXM assay was employed to assess for AECAs using fixed preparation made from human pulmonary microvascular ECs. The results were visualized with IIF with goat anti-human IgG Abs	IIF is a less specific approach compared with other detection methods such as flow cytometry; only one pre-transplant serum sample was assessed for AECAs; biopsy data showed evidence of prior microbial infections that may have enhanced the alloantigenic response
Reinsmoen et al., 2017 (29)	Lung	In LTRs with pre-transplant HLA-DSA, higher frequencies for either AT_1_R-Abs or ETAR-Abs correlated with increased potential to develop *de-novo* DSA	The pre- and post-transplant sera of 162 LTRs were tested for anti-AT_1_R and ETAR-Abs	Commercially available sandwich ELISAs were used to detect anti-AT_1_R and ETAR-Abs	ECXMs were not performed to assess the impact of other AECAs; relatively short follow-up time (3–6 months post-transplant samples were tested)
Otten et al., 2006 (30)	Lung	The SEREX technique identified six potential non-HLA targets that were shared between four study patients	Pre- and post-transplant serum samples from 11 LTRs were tested for non-HLA	SEREX technique was used to test for reactivity against the cDNA library-encoded antigens expressed by the transfected bacteria, and this reactivity was visualized by a goat anti-human IgG	Small sample size; the assay failed to detect gene products in the pre-transplant samples; the assay only detected antigenic targets, but their specific role in rejection was not established

AECAs, anti-endothelial cell antibodies; AT_1_R, angiotensin type 1 receptor; cDNA, complementary DNA; ECs, endothelial cells; ETAR, endothelin type A receptor; ECXM, endothelial cells crossmatching; IIF, indirect immunofluorescence; LTR, lung transplant recipient; SEREX, serological analysis of recombinant cDNA expression libraries.

a Sample details as well as the type of assay pertain only to the non-HLA detection method described in the article and not the entire methodology section.

**Table 3:** Non-HLA antibody detection studies in liver transplantation.

Reference	Organ	Overall conclusion	Sample details^a^	Type of assay^a^	Key limitations
Ekong et al., 2019 (41)	Liver	With regard to non-HLA Abs, no significant association was found between anti-nuclear Abs, anti-smooth muscle Abs, anti-liver kidney microsomal Abs, and AT_1_R Abs on the development of fibrosis	Serum samples from 42 recipients were assessed for AT_1_R-Abs and several other non-HLA Abs	Antinuclear Abs were measured by IIF using an IgG-specific conjugate; anti-smooth muscle and anti-liver kidney microsome Abs were measured using a semiquantitative ELISA; and AT_1_R-Abs were measured using commercial ELISA	Non-HLA Abs data were missing for a number of patients pre-transplant; exclusion of some patients due to lack of HLA-DSA measurement post-transplant reduced the sample size of the study; the study mostly focused on HLA-DSA and little attention was given to non-HLA Abs
Ohe et al., 2014 (42)	Liver	All patients with increased levels for both HLA-DSA and AT_1_R-Abs were found to have advanced fibrosis compared with the other groups positive for either Ab or negative for both	Post-transplant sera from 81 patients were screened for AT_1_R-Abs	AT_1_R-Abs were detected in the sera using a commercially available ELISA kit	Only patients withdrawn from immunosuppression treatment were included; no assessment of Abs after the reintroduction of the immunosuppression medication; due to limited post-transplant serum samples, the status of preformed or *de-novo* Abs was not established
O’Leary et al., 2017 (43)	Liver	Preformed non-HLA Abs alone did not impact the clinical outcomes; however, the synergistic association between these preformed Abs and HLA-DSA increased the mortality risk significantly	Pre- and post-transplant serum samples from 1,269 liver transplant recipients were analyzed for anti-AT_1_R or ETAR-Abs	Commercially available sandwiched ELISA kits were used to measure the levels of anti-AT_1_R or ETAR-Abs	This study was retrospective and single centered; only the association between non-HLA Abs and HLA-DSA and the development of fibrosis could be established
Xu et al., 2021 (14)	Liver	Among all tested autoantibodies, patients with Abs against LG3 experienced worse secondary graft survival compared with those without. The combination of LG3 with AT_1_R or HLA-DSA showed a higher rejection risk	Pre-transplant sera of 131 transplant recipients who received a second liver were tested for 33 autoantibodies	A commercially available Luminex antibody panel was used to screen for the presence of non-HLA Abs	No post-transplant serum samples were assessed for the changes in the levels of the preformed Abs after transplantation and the development of *de-novo* Abs

AT_1_R, angiotensin type 1 receptor; ETAR, endothelin type A receptor; IIF, indirect immunofluorescence; LG3, C-terminal laminin-like globular domain of perlecan; non-HLA Abs, non-human leukocyte antigen antibodies.

a Sample details as well as the type of assay pertain only to the non-HLA detection method described in the article and not the entire methodology section.

**Table 4:** Non-HLA antibody detection studies in heart transplantation.

Reference	Organ	Overall conclusion	Sample details^a^	Type of assay^a^	Key limitations
Hiemann et al., 2012 (44)	Heart	Increased levels of anti-AT_1_R and ETAR-Abs were present in patients experiencing both ACR and ABMR compared with patients with no rejection. Increased pre-transplant titers of these Abs were associated with a higher risk for an early onset of microvasculopathy	Pre- and post-transplant serum samples from 30 patients were assessed for the presence of both anti-AT_1_R and ETAR-Abs	Commercially available ELISAs were used to measure anti-AT_1_R and ETAR-Abs levels	Small sample size; a large number of patients in this study were on assist device support which represents a high-risk group for sensitization events; no associations were established with regard to pre-transplant HLA-DSA
Jurcevic et al., 2001 (13)	Heart	Using the in-house ELISA, a predictive test for the development of CAD was established with 63% sensitivity and 76% specificity based on the mean titer of the vimentin-Abs detected in the first 2 years after the transplant	Pre- and post-transplant serum samples from 109 patients were assessed for vimentin-Abs	An in-house developed ELISA kit was used to measure anti-vimentin IgM levels in the serum	The majority of the post-transplant serum samples were collected 2 years after transplantation even though the patients were followed for 5 years; therefore, the data from the first 2 years only were analyzed with regard to the titers of vimentin-Abs and CAD
Zhang et al., 2011 (45)	Heart	The assay showed that each tested patient had at least one non-HLA Ab identified, with vimentin-Abs being the most frequent in this patient group	Post-transplant serum samples from 13 patients with treated ABMR and/or ventricular dysfunction and without HLA-DSA were screened for 32 non-HLA Abs	The non-HLA Abs were detected using a commercial Luminex kit with fluorescence-labeled secondary anti-human IgG	The small sample size made it difficult to establish associations between vimentin-Abs and ABMR in the pre-transplant sera; variations in time between the non-HLA testing and the time the biopsies were taken due to follow-up of patients from another center
Butler et al., 2020 (46)	Heart	18 non-HLA Abs associated with rejection were identified, among which, 4 Abs were not previously described as non-HLA targets. Within the 18 identified non-HLA Abs, 5 of them predicted rejection, and 4 of showed a synergistic effect with HLA-DSA	546 serum samples from 115 heart transplant recipients were screened for non-HLA antibodies	A commercial multiplex bead array that included 67 non-HLA targets was used. Antibody binding was reported as the MFI of IgG with Luminex	No pre-transplant serum samples were tested; important non-HLA antibodies such as AT_1_R could not be included in the Luminex panel

ABMR, antibody-mediated rejection; ACR, acute cellular rejection; AT_1_R, angiotensin type 1 receptor; CAD, transplant-associated coronary artery disease; ETAR, endothelin type A receptor; MFI, median fluorescence intensity; non-HLA Abs, non-human leukocyte antigen antibodies.

a Sample details as well as the type of assay pertain only to the non-HLA detection method described in the article and not the entire methodology section.

**Table 5:** Non-HLA antibody detection studies in composite tissue transplantation.

Reference	Organ	Overall conclusion	Sample details^a^	Type of assay^a^	Key limitations
Banasik et al., 2014 (47)	Hand	The repeated occurrence of rejection episodes was associated with high levels of anti-AT_1_R and ETAR-Abs in one patient with bilateral hand transplantation	Post-transplant serum samples from six patients were assayed for AECAs and anti-AT_1_R and ETAR-Abs	AECAs were detected with the TITERPLANE technique using HUVECs, and the reaction of the antigen to IgG, IgA, or IgG was visualized with IIF. Antibodies against AT_1_R and ETAR were detected with the commercial ELISAs	The study only included five patients; only post-transplant serum samples were assessed for non-HLA Abs
Sikorska et al., 2022 (48)	Hand	Repeated episodes of rejection were associated with high levels of anti-AT_1_R and ETAR Abs, as well as increased levels of EC activation in one out of the six included patients. No elevations in pro-inflammatory cytokines (IL-1, IL-6, IFNγ) were observed	Post-transplant sera from six hand transplant recipients were assayed for anti-AT_1_R, ETAR, PAR-1, and VECGF-A Abs. Proinflammatory cytokines (IL-1, IL-6, IFNγ) were also assayed to evaluate the humoral response post-transplant	Anti-AT_1_R, ETAR, PAR-1, and VECGF-A Abs using commercial ELISAs. Proinflammatory cytokines (IL-1, IL-6, IFNγ) were also assayed with ELISAs	A small sample size of six patients; no pre-transplant samples were analyzed; unclear how the ELISA protocols were conducted or whether the kits were made in-house or acquired commercially

AECAs, anti-endothelial cells antibodies; AT_1_R, angiotensin type 1 receptor; ECs, endothelial cells; ETAR, endothelin type A receptor; IIF, indirect immunofluorescence; IFNγ, interferon gamma; IL-1, interleukin 1; IL-6, interleukin 6; non-HLA Abs, non-human leukocyte antigen antibodies; PAR-1, protease-activated receptor 1; VECGF-A, vascular endothelial growth factor A.

a Sample details as well as the type of assay pertain only to the non-HLA detection method described in the article and not the entire methodology section.

## Non-HLA Antibodies in Kidney Transplantation

Cell-based crossmatching assays were among the very first techniques used to detect possible non-HLA antibodies in the serum samples from renal transplant patients (21, 24, 27, 40, 49–51). In these assays, primary endothelial cells (ECs) are used as targets to be tested against the recipients’ sera. These *in-vitro* assays are considered to be relatively easy to perform, cost-efficient, and not labor intensive. In addition, it has been suggested that these assays could also detect antibodies against polymorphic antigens such as AT_1_R and MICA which might differ between donors, making their use in the clinical setting more justified.

In kidney transplantation, cell-based crossmatching assays have been used to screen for AECAs. In fact, the use of ECs as targets for crossmatches with fluorochromasia as the reporting technique has already existed since the late 1980s (49). An indirect immunofluorescence procedure was also described by Pontes and colleagues in 2001 where they showed that cultured human umbilical cord vein endothelial cells (HUVECs) could be potentially used to detect non-HLA antibody reactivity in kidney transplant recipients (KTRs). They tested the serum from a single patient who experienced rejection due to antibodies directed against endothelial antigens that were not expressed on platelets (i.e., non-HLA antigens). To ensure that the assay was specific for AECA, they used a platelet pool to remove all pre-existing HLA class I antibodies and screened for reactivity against HLA class I with a complement-dependent cytotoxicity (CDC) assay resulting in a negative CDC test (32).

Ming et al. made use of HUVECs for a retrospective crossmatching assay with flow cytometry (33). The assay was used to test whether the study patient that presented with acute ABMR, who had received kidney transplant without evidence of HLA-DSA, was in fact due to anti-MICA antibodies (33). High levels of antibodies against MICA were observed using a commercial MICA single antigen Luminex bead assay in both the pre- and post-transplant serum samples, and the donor specificity of these antibodies was confirmed with Sanger sequencing of both the donor and recipient MICA genotype. Finally, endothelial cell-based crossmatching with HUVECs was used to further confirm and characterize the MICA antibodies. The assay demonstrated binding and cytotoxic effects of MICA-DSA in the recipient serum on HUVECs, which indicated the expression of the antigens on the surface of the endothelium and provided evidence that the ABMR experienced by the patient after the first transplantation could have been attributed to MICA-DSA (33).

Crespo et al. recently published a paper in which they described a systematic exploration of pre-and post-kidney transplantation sera for HLA and non-HLA antibodies (34). One hundred and eighteen kidney transplant recipients were included, based on histological pathology scored as normal histology, interstitial fibrosis and tubular atrophy (IFTA), and ABMR based on Banff’15. The biopsies were either surveillance or clinically indicated biopsies taken after ABO-compatible kidney transplantation with a negative CDC crossmatch. HLA antibodies were detected using the Luminex HLA single antigen bead assay and MICA was also detected using Luminex technology. AT_1_R-Ab and ETAR-Ab were measured using commercially available ELISAs (34). They additionally performed EC crossmatch (ECXM) assays, using primary human aortic endothelial cells isolated from aortic rings of explanted donor hearts (45). They found that the combination of pre-transplant HLA-DSA and AT_1_R-Abs was strongly associated with ABMR histology. Both pre-transplant DSA and AT_1_R-Abs were significantly associated with the development of ABMR. However, none of the patients with HLA-DSA-negative ABMR had AT_1_R-Abs. In addition, the post-transplant combination of HLA-DSA and AT_1_R-Abs did not associate with the development of ABMR. Furthermore, neither pre-/post-transplant MICA- and ETAR-Abs nor a positive ECXM correlated with ABMR histology, with or without HLA-DSA. Positivity in the ECXM was found in all different patient groups and did not associate with any histological signs of endothelial damage, e.g., ABMR histology.

Although the previously mentioned studies made use of primary ECs as targets for the crossmatching test, these cells were not derived from the particular graft donor. An EC-based crossmatching assay with donor-derived ECs may provide a better risk assessment for the recipients. However, difficulties in obtaining cell cultures of organ-specific endothelial subsets have hampered molecular characterization, transcriptional profiling, and assay development with ECs. Nevertheless, isolation of ECs from donors is possible. Although the cell isolation can be time-consuming and expensive, it is a promising technique that will allow us to study donor-specific HLA and non-HLA antibody-dependent endothelial cytotoxicity (52). One of the earliest approaches that achieved donor-derived endothelial cell crossmatching test was introduced in 2002 by Vermehren and colleagues (50), which consequently resulted in the development of the commercially available flow cytometry-based assay XM-ONE^®^ (24).

The proprietary XM-ONE assay is designed to screen for non-HLA antibodies in a crossmatching test that uses endothelial precursor cells (EPCs) as target cells selected by the expression of angiopoietin receptor (Tie-2+). The assay was validated in a multicenter trial by Breimer et al. that used EPCs isolated from donor peripheral blood mononuclear cells (PBMCs) to screen for AECAs in the pre-transplant serum samples of 147 patients (21). The trial identified AECAs in 35 of the 147 included patients (24%), among which a significant number (16 of 35; 46%) either experienced rejection (up to 3 months after transplantation) or were at a higher risk of impaired kidney function (as indicated by the increased serum creatinine levels) compared with those without AECAs (13 of 112; 12%) (21).

Several other studies utilized the XM-ONE setup to screen for the presence of non-HLA antibodies in living donor kidney transplant recipients. These studies describe contrasting results (26, 27). Soyöz et al. screened for EPC-reactive IgG and IgM in post-transplant serum from 13 living donor transplant recipients using the XM-ONE kit. In this population, AECAs were not detectable in the serum samples of all patients including the three patients who experienced ABMR (26). In addition, Zitzner et al. also investigated the presence of AECAs in the pre-transplant serum samples of 150 living donor kidney transplant recipients and reported a lack of association between the XM-ONE result and biopsy-proven rejection or vasculopathy at 1-year post-transplant (27).

The XM-ONE assay has several limitations. For instance, the properties of the circulating EPCs might not be reflective of the ECs in the transplanted allograft. It is essential to keep this in mind, especially since these precursor cells lack important general EC markers such as CD31 and CD34 (50). Additionally, Tie-2+ EPCs express HLA class I and class II antigens which can lead to false positivity in the presence of HLA-DSA and would require the depletion of HLA-DSA from the recipient’s serum prior to testing (50). Furthermore, Alheim and colleagues reported that a large fraction of the cells isolated with the XM-ONE kit were lymphocytes positive for the Tie-2 receptor (24). This would require further gating for CD3+ CD19+ lymphocytes to exclude the possibility of contamination and interference of Tie-2+ lymphocytes when performing the endothelial crossmatching test. Our group recently published a method of obtaining human renal ECs from machine-perfused donor kidneys. We demonstrated that these cells expressed common EC markers (CD31, CD34, von Willebrand factor, VEGFR-2, PV-1, and HLA-DR to a variable extent). As these ECs are derived from the site of the graft, they could potentially be better candidates as targets for an endothelial crossmatching assay (52). Delville et al. recently demonstrated that non-HLA antibodies that are associated with the histology of ABMR primarily bind in a very specific manner to glomerular endothelial cells (CiGEnCs) (53). However, the basal expression of HLA antigens limited the application of CiGEnCs for non-HLA antibody detection in patients without circulating anti-HLA antibodies. To tackle this obstacle, the group applied a CRISPR/Cas9 strategy to delete the B2M and CIITA genes, resulting in loss of function and undetectable HLA-ABC and HLA-DR expression (40). Using these cells, a non-HLA antibody detection immunoassay (NHADIA) was developed. The authors used an unselected cohort of kidney transplant recipients and showed that non-HLA antibodies were increased in patients who underwent previous kidney transplantation. The pre-transplantation NHADIA value correlated with microvascular inflammation (MVI) in the kidney allograft at 3 and 12 months post-transplantation and was correlated with the risk of the developing ABMR. Interestingly, no correlation between NHADIA results and AT_1_R levels was found, indicating that the antibodies detected in the NHADIA results are not AT_1_R antibodies. The results from these studies suggest that non-HLA antibodies associated with the histology of ABMR which bind to CiGEnCs might recognize a wide diversity of antigens. In addition, these results point directly toward the major limitation of using a single cell line for endothelial cell crossmatching assays, as a single cell line does not address the variability of expressed antigens between individuals, which may form the basis for non-HLA antibody formation.

The endothelium has different functions corresponding to the region of the body where it is situated (54). This results in different antigen expression patterns, and therefore, ECs may respond differently to activation (55, 56). The importance of using ECs derived from vessels of the appropriate location has been underlined by several studies. This includes work on AECAs in various small and large vessel diseases and responses of ECs from different organs to inflammation and sepsis (55–58). Moreover, it was shown that upon binding of HLA class I antibodies on human-aortic, umbilical, and dermal microvasculature ECs, the induction of P-selectin, involved in the recruitment of leukocytes, varied between EC types (59). Some studies use immortalized endothelial cell lines instead of primary cells for their assays. A comparison of HUVECs and EA.hy926 (a commonly used immortalized HUVEC cell line) revealed that EA.hy926 cells have a high similarity with primary ECs; however, they show differences in the expression levels of certain EC markers. They also express a large number of additional genes mainly related to the cell cycle and EC apoptosis (59). Circulating EPCs isolated from peripheral blood allow testing for donor-specific AECAs. However, it is not clear whether these cells reflect the properties of the endothelial cells present in the graft (59). Therefore, antibodies reactive against antigen targets on EPCs or HUVECs may not react against antigens expressed on renal microvascular endothelial cells. Li et al. underlined that non-HLA antibodies are not exclusively directed against targets on the endothelium, by testing pre- and post-transplant samples of pediatric renal transplant patients for reactivity against over 5,000 protein targets selected based on their appearance in the kidney using a ProtoArray. They reported a response to 61% of the targets on average with the highest reactivity against antigens expressed on pelvic epithelial cells and in the renal cortex (38). In addition, two separate studies by Leisman and Lammerts et al. recently showed that AT_1_R is possibly not expressed by renal endothelial cells, and Delville et al. did not find a correlation between AT_1_R and non-HLA anti-endothelial cell antibodies, measured with the NHADIA (52, 53, 60). Also, the study by Senev et al. reported that AT_1_R antibodies assessed using the multiplex Luminex assay could not explain the histology of ABMR in the absence of DSA (22).

In addition to cell-based crossmatching assay, antigen detection methods have also been used to screen for known specific non-HLA antibodies. ELISAs are universally used for convenient and rapid bulk screening of patient serum samples, which is further eased by the possibility of acquiring some of the kits commercially. For instance, currently, there are two commercially available ELISA kits that have been developed by CellTrend (Luckenwalde, Germany) supplied by One Lambda, for detecting anti-AT_1_R or anti-ETAR antibodies in kidney transplant patients (11, 12, 34, 35, 61, 62). Unlike conventional ELISAs whereby the proteins of interest are detected by antibodies from a purified or homogeneous denatured cell-lysate samples, the commercially available AT_1_R and ETAR ELISAs are produced by coating the plate with non-denatured extracts from cells overexpressing the target protein. This ensures that the detected targets (i.e., antibodies against AT_1_R and ETAR) are complementary to the receptors in their native, non-denatured form (62).

Several researchers utilized these commercial ELISAs to investigate the relationship between AT_1_R or ETAR and the clinical outcomes in renal transplant recipients (11, 12, 35). Philogene et al. investigated the presence of AT_1_R antibodies with a commercial ELISA in combination with XM-ONE (for AECAs) in the post-transplant sera of kidney transplant recipients who had low or negative HLA-DSA. They observed an association between the development of ABMR and the levels of AT_1_R antibodies especially when HLA-DSA were detected (12). The XM-ONE results revealed that patients whose post-transplant sera were positive for AECAs had increased AT_1_R titers compared with those with the negative crossmatching test. Interestingly, 8 out of the 11 patients with positive XM-ONE test developed rejection, 1 developed transplant glomerulopathy, and 2 had no rejections. Similarly, Pearl et al. also reported a strong association between AT_1_R and ETAR antibodies, microvascular injury, elevated levels of IL-8, and impaired kidney function upon investigation of AT_1_R and ETAR antibody levels in the post-transplant serum samples of kidney transplant recipients using commercial ELISAs (11).

More recently, Yu and colleagues used the anti-AT_1_R antibody ELISA to evaluate their role in predicting the transplant outcome in low-risk, living donor KTRs (31). The study included 94 transplant recipients who had negative pre-transplant serum HLA-DSA who underwent ABO-compatible, living donor kidney transplantation. In this study, anti-AT_1_R antibody titers were measured in 94 pre-transplant serum samples and 29 post-transplant serum samples in patients experiencing biopsy-proven rejection. A significant association was found between increased pre-transplant serum levels of anti-AT_1_R antibodies and the risk for developing acute rejection. Interestingly, the authors also investigated the presence of other AECAs in the pre-transplant sera with the XM-ONE assay. They reported that patients with a positive XM-ONE AECA test had poorer kidney function, starting 3 months after transplantation and continuing to decline up until 20 months (end of the study) (31).

Aside from ELISA, genome-wide analyses and protein microarrays have also been described for antigen identification (36–38). Using genome-wide analysis, Reindl-Schwaighofer and colleagues were able to show that genetic mismatches of non-HLA haplotypes coding for transmembrane or secreted proteins were associated with an increased risk of functional graft loss (36). They genotyped 477 KTRs receiving their first kidney transplant from a deceased donor, and genetic mismatches between the pairs were measured to identify incompatibilities in both transmembrane and secreted proteins. From this, they were able to identify 16 non-HLA donor-specific peptide mismatches which they then used to construct an array of peptides to screen another group of 25 patients with biopsy rejection for the presence of these mismatches. The study showed that mismatches of non-HLA were associated with worse clinical outcomes independent of HLA (36).

In protein microarrays, thousands of recombinant proteins are immobilized and arranged on a solid surface and are then probed with a fluorescent-labeled sample. Protein microarrays can be used for various applications, including antibody detection (63).

Jackson et al. employed ProtoArray^®^ (a protein microarray technology), in combination with ELISAs, to identify target antigens for AECAs. The sera of 10 KTRs from a discovery cohort experiencing ABMR in the absence of HLA-DSA were used as samples for the protein array assay (37). The assay identified four antigenic targets, namely, endoglin, Fms-like tyrosine kinase-3 ligand (FLT3), EGF-like repeats and discoidin I-like domains 3 (EDIL3), and intercellular adhesion molecule 4, all of which have been shown (*in vitro*) to be capable of endothelial cell activation and induction of pro-inflammatory cytokines and chemokines. To further validate these findings, an additional 150 pre- and post-transplant serum samples from KTRs were tested for antibodies against these antigens with in-house-made ELISAs, and positive results were observed in 24% of the tested pre-transplant samples. The presence of antibodies against these antigens was associated with HLA-DSA sensitization, ABMR, and early transplant glomerulopathy (37).

Li et al. utilized the ProtoArray technology to specifically investigate the antibody response against HLA and MICA to determine whether the immunogenic response in the kidney tissue was restricted to certain compartments that express these antigens (38). Pre- and post-transplant serum samples from 18 pediatric kidney transplant recipients were examined for non-HLA antibody response. The protein microarray assay revealed an increase in the signal for *de-novo* antibodies by an average of 61% in all patients in the post-transplant serum compared with the pre-transplant. Of note, anti-MICA antibodies were detected in 72% of the patients post-transplant (38). The authors did not test for any correlation between the development of the anti-MICA antibodies and the risk of rejection in the recipients even though previous studies have reported the existence of such correlations (64, 65).

Moreover, a Luminex-based non-HLA antibody assay was developed covering 14 possible targets for non-HLA antibodies (39). Kamburova and colleagues constructed a multiplex Luminex assay with 31 different microspheres consisting of various proteins to screen the serum of kidney transplant recipients for 14 non-HLA antibodies. Various testing conditions were used in order to optimize and validate the highest specific median fluorescence intensity (MFI) levels that could distinguish between the positive and negative patient sera. The authors reported that anti-phospholipase A2 receptor (PLA2R) antibodies showed the highest level of distinction (in terms of MFI vs. background signal) followed by anti-vimentin antibodies. Although the assay did not investigate the presence of commonly described non-HLA targets such as AT_1_R and ETAR, the multiplex assay was used in a subsequent study to determine the non-HLA status in the pre-transplant serum samples of 4,770 patients in the PROCARE cohort (8). The analysis revealed that antibodies against rho GDP dissociation inhibitor beta (ARHGDIB) were more clinically relevant in patients receiving a deceased donor kidney transplant compared with those receiving a living donor kidney transplant. Additionally, this non-HLA antibody seemed to be associated with negative clinical outcome on the graft that was independent of the HLA-DSA status. These results were confirmed in a smaller cohort by Senev et al. (22) Interestingly, these results of a negative clinical outcome when non-HLA antibodies are present in the pre-transplant sera were similar to what has been published by other groups for other types of non-HLA antibodies [namely, the studies by Soyöz et al. and Zitzner et al. (26, 27)]. This especially holds true with regard to the presence and clinical relevance of non-HLA antibodies in living donor compared with deceased donor transplantation.

**Figure 2:**
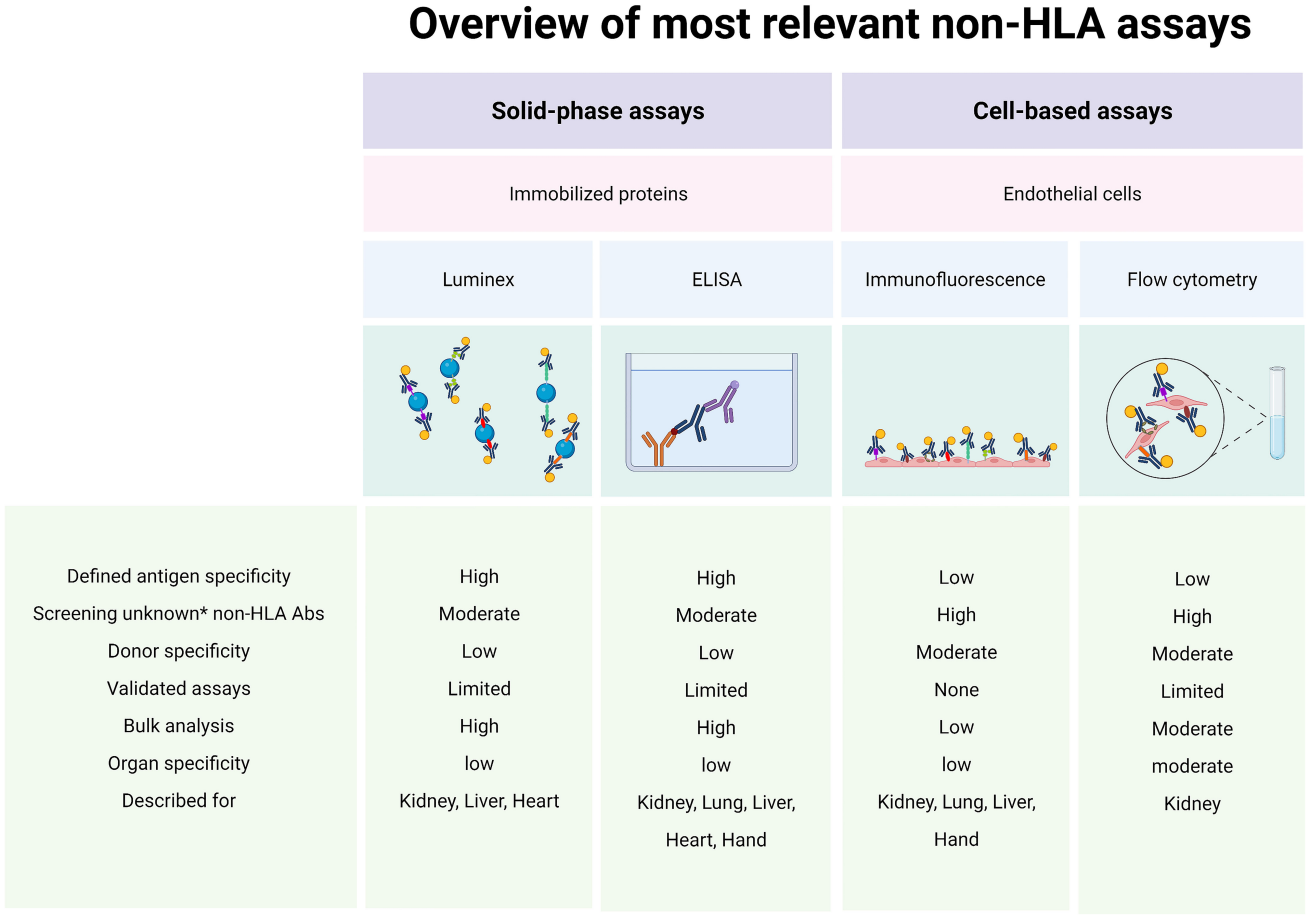
Assays for alloreactive antibodies. Abs, antibodies; ELISA, enzyme-linked immunosorbent assay; HLA, human leukocyte antigen. * New/unknown non-HLA Abs. Created with biorender.com.

The bottom line is that we now possess several relevant cell-based assays (cytotoxic assays or flow cytometric crossmatch assays) and solid-phase assays (Luminex, enzyme-linked immunosorbent assays) that can detect or screen for potentially alloreactive antibodies (**Figure 2**). Several studies demonstrated that pre-transplant non-HLA antibodies have pathogenic effects on graft survival and may contribute to impaired kidney function post-transplant (8, 10, 21, 31, 36). Others showed that this only holds true when HLA antibodies were also present (9, 11, 34, 37, 38). Moreover, AT_1_R antibodies are the most studied non-HLA antibodies despite evidence suggesting that they are not expressed by the involvement of ECs in the kidney tissue (52, 60). Importantly, the large variety of non-HLA antibodies used as targets for investigation in the different studies makes it difficult to compare or predict the exact role of these antibodies. In addition, although the majority of studies use IgG as readout, many studies report non-HLA antibodies with variable readouts (IgG, IgM, C1q). In the future, it will be important that studies use a comparable and reproducible approach with well-defined study populations and endpoints and thoroughly validate their assays, addressing assay sensitivity and specificity, intra- and interassay variability, positive thresholds, and controls.

## Non-HLA Antibodies in Lung Transplantation

While several research groups in the field of kidney transplantation have utilized cell-based crossmatching assays to screen for non-HLA targets, this technique is rarely described in lung transplantation research.

Margo and colleagues described an indirect immunofluorescence cell-based crossmatching assay to detect the presence of AECAs in the serum samples of lung transplant patients (28). Cytocentrifuged fixed preparations made from human pulmonary microvascular EC cultures were used as the target cells for testing. The study population was tested for panel-reactive antibodies (PRA) and was found to be negative before transplantation and at the time of rejection. The indirect immunofluorescence assay revealed a prominent granular nuclear and cytoplasmic staining pattern indicative of positive reactivity to endothelial cells in 18 of 19 patients (28). It is important to mention that this study was not explicitly designed to demonstrate the potential role of EC antigens as the antigenic targets; rather, the authors’ original goal was to present a report of a series of lung patients who had developed post-transplant septal capillary injury syndrome of humoral origin as a form of allograft rejection. Furthermore, indirect immunofluorescence is less sensitive compared with newer detection techniques such as flow cytometry which essentially limits the significance of these findings.

In addition to cell-based assays, antigen detection techniques such as ELISA (29) and SEREX (30) have also been described in the context of lung transplantation.

Reinsmoen et al. investigated the presence of antibodies to AT_1_R and ETAR in the pre- and post-transplant sera of 162 lung transplant recipients from three centers with a commercial sandwich ELISA kit (29). The serum samples were tested for binding to AT_1_R and ETAR at three levels: strong, intermediate, and negative. For AT_1_R, the frequencies of bindings were 46%, 37%, and 17%, respectively, while ETAR had 26%, 29%, and 45% binding frequencies. Stronger binding frequencies for both AT_1_R and ETAR were significantly correlated with increased potential to develop ABMR post-transplant. However, out of the 162 recipients tested, only 5 developed ABMR post-transplant, and these 5 patients were among those who developed *de-novo* DSA.

The SEREX technique is an antigen identification technique that uses cDNA libraries extracted from solid tissue screened against sera from patients to identify gene products [usually expressed by a bacterium such as *Escherichia coli* (*E. coli*) transfected with the genes of interest], which are then recognized by an IgG antibody (66). Although mainly used to identify tumor antigens, this technology was applied to lung transplantation to better understand the role of non-HLA antibodies in the pathogenesis of bronchiolitis obliterans syndrome (BOS) in a study by Otten and colleagues (30).

In this study, RNA from the airway epithelial cells was obtained from the tracheae of four donors and was used to construct a complementary DNA (cDNA) library which was then transfected to *E. coli*. Pre- and post-transplant serum samples from 11 lung recipients were tested for reactivity against the antigens expressed by the transfected bacteria, and the reactivity of the sera was visualized by a goat anti-human IgG. The assay identified six non-HLA targets that were only shared between four of the study patients confirming that the non-HLA profile differs among individuals, and therefore, a larger cDNA library might be needed to cover a wider range of specific non-HLA that might contribute to the development of BOS in the transplant population (30). The results of this study, despite the relatively smaller sample size, demonstrated that the SEREX technique was capable of identifying potential non-HLA targets present after lung transplantation, and the authors suggest a potential role of this technique to be implemented for clinical testing (30).

In conclusion, non-HLA antibodies may contribute to the pathogenesis of lung transplant rejection, but their specific targets have yet to be identified (**Figure 2**). Even with elaborate technologies utilizing cDNA libraries or validated solid-phase assays (i.e., AT_1_R and ETAR ELISAs), the exact contributions of non-HLA to graft dysfunction in lung transplantation is still unknown.

## Non-HLA Antibodies in Liver Transplantation

Liver transplant recipients rarely experience ABMR even in the presence of antibodies directed against HLA (43). Although HLA antibodies have been gaining attention for their association with adverse transplantation outcomes, research into non-HLA antibodies in liver transplant patients is still relatively new and data are limited. In fact, most of the studies into the role of non-HLA antibodies focused primarily on the detection of AT_1_R and ETAR antibodies.

Ekong and colleagues evaluated the effects of several non-HLA antibodies including anti-nuclear antibodies, anti-smooth muscle antibodies, anti-liver kidney microsomal antibodies, and AT_1_R antibodies on the development of fibrosis in 42 pediatric liver transplant recipients using indirect fluorescence and a commercially available ELISA kit (41). The results of their analysis revealed that the presence of the aforementioned antibodies had no significant association with fibrosis. That said, the study mainly focused on evaluating whether HLA epitope mismatches were predictors of *de-novo* donor DSA risk and rejection and less on the role of non-HLA antibodies specifically (41). Moreover, most of the patients did not have pre-transplant serum samples available for testing for anti-AT_1_R antibodies, so the levels of this antibody were not clearly defined in these patients pre-transplant.

Ohe and colleagues also focused on evaluating the effects of anti-AT_1_R antibodies in a cohort of 81 pediatric living donor liver transplant recipients using a commercially available ELISA kit (42). The study concluded that all patients who had anti-AT_1_R antibodies in addition to HLA-DSA developed advanced fibrosis compared with those patients who only had one antibody or were double negative for both. Assessment of the AT_1_R status especially in patients with confirmed DSA could prove to be useful in predicting the risk for fibrosis. However, even though it was clear that the AT_1_R antibodies played an important role in fibrosis in liver transplant recipients, the results only suggested an association between these antibodies and the development of fibrosis and not a causation. Interestingly, these findings resemble those reported by Crespo in kidney transplantation as discussed above.

In a larger cohort of adult liver transplant patients, O’Leary et al. reported that AT_1_R or ETAR antibodies (either preformed or *de novo*) were associated with a higher risk of rejection (43). They analyzed pre- and post-transplant serum samples from 1,269 liver transplant recipients for AT_1_R or ETAR antibodies using commercially available sandwiched ELISA kits. The results showed that non-HLA antibodies alone did not influence the outcome of the transplant; however, when coupled with an HLA-DSA (particularly of the IgG3 subclass), the synergistic association between these antibodies increased the mortality risk significantly (hazard ratio, 1.66; *P* = 0.02). Additionally, they reported that post-transplant non-HLA antibodies were capable of activating the complement system as seen from the positive C4d staining pattern in the liver tissue.

In addition to AT_1_R and ETAR antibodies, the C-terminal laminin-like globular domain of perlecan (LG3) antibodies was also investigated in liver transplantation. Xu and colleagues recently published a study in which they tested the pre-transplant sera of 131 transplant recipients who received a second liver for 33 autoantibodies with a commercially available Luminex antibody panel (14). Among these 33 antibodies, 15 were significantly higher in 52% of the patients who lost their graft. Specifically, patients with antibodies against LG3 experienced worse secondary graft survival compared with those without this particular antibody (*P* = 0.02). Interestingly, patients with increased AT_1_R antibody levels in addition to LG3 were at a higher risk for rejection compared with those with either of these antibodies. A similar association was found between LG3 levels and HLA-DSA, which, once again, suggested a synergistic relationship. Therefore, screening for LG3 (in addition to AT_1_R and ETAR antibodies) might be important for liver transplant recipients with or without HLA-DSA as it may significantly help in identifying high-risk transplant patients.

In summary, while allograft rejection is relatively rare in liver transplantation due to the highly immunotolerant nature of the organ (43), research into the role of non-HLA antibodies, particularly anti-AT_1_R and ETAR antibodies in liver transplant patients, is gaining the attention of researchers. However, as it stands, research in this area is still relatively new and the reported data are limited (**Figure 2**).

## Non-HLA Antibodies in Heart Transplantation

In heart transplantation, the detection of non-HLA antibodies still remains a major endeavor, especially when it was reported that 40% of patients experiencing biopsy-proven ABMR had no HLA-DSA in blood (45). Investigation into non-HLA antibodies in this field mainly focuses on the detection of the specific non-HLA, especially the antigens expressed on the endothelium, utilizing approaches such as ELISAs and Luminex.

Hiemann and colleagues utilized both AT_1_R and ETAR ELISAs to investigate the impact of anti-AT_1_R and anti-ETAR antibodies on the development of ABMR in heart transplant recipients (44). They prospectively assessed the pre- and post-transplant serum samples from 30 patients for the presence of both anti-AT_1_R and anti-ETAR antibodies with commercially available sandwiched ELISA kits. The results showed elevated levels of anti-AT_1_R and anti-ETAR antibodies present in patients experiencing both cellular and ABMR compared with patients with no rejection. Furthermore, increased pre-transplant titers of these antibodies were associated with a higher risk for an early onset of microvasculopathy, implying negative effects post-transplant (44).

In addition to using ELISAs for AT_1_R and ETAR, Jurcevic et al. developed an ELISA for detection of anti-vimentin antibodies (13). Pre- and post-transplant serum samples from 109 cardiac transplant recipients were tested for anti-vimentin antibodies up to 5 years after transplantation, and the antibody titers were correlated to the development of transplant-associated coronary artery disease. The mean titers of anti-vimentin antibodies calculated in the period between 1 and 5 years post-transplant were significantly increased in patients who had developed transplant-associated coronary artery disease compared with those free from the disease. Additionally, the assay also helped establish a predictive test for the development of this disease with 63% sensitivity and 76% specificity based on the mean titer of the antibodies detected in the first 2 years after the transplant. Therefore, by utilizing this ELISA, anti-vimentin antibodies could potentially be used as biomarkers for identifying patients who have a higher risk for developing transplant-associated coronary artery disease (13).

Luminex technology for the screening of non-HLA antibodies in heart transplantation has also been described. In their study, Zhang and colleagues employed a multiplex bead panel to profile non-HLA antibodies in heart transplant recipients with treated ABMR (67). Post-transplant serum samples from 13 patients with treated ABMR and/or ventricular dysfunction and without HLA-DSA were screened for 32 non-HLA antibodies with a commercially available panel. They were able to show that each tested patient had at least one non-HLA antibody identified, with anti-vimentin antibodies being the most frequent in the patient group with treated ABMR with undetectable HLA-DSA. Additionally, they also examined pre-transplant serum samples for anti-vimentin antibodies, and the analysis revealed that 11 out of the 13 study patients were negative for vimentin pre-transplant; however, in 7 of these patients, anti-vimentin antibodies were detected at the time of ABMR, suggesting a *de-novo* development of these antibodies post-transplant (67).

Butler et al. also utilized a commercialized Luminex-based multiplex bead panel for the discovery of non-HLA antigens associated with heart transplant rejection (46). First, a protein microarray was constructed to identify 366 non-HLA targets from a discovery cohort consisting of 12 heart transplant recipients who had positive endothelial cell crossmatch but no evidence of HLA-DSA at the time of biopsy-proven rejection. A commercial multiplex bead array that included 67 non-HLA targets was then used to screen 546 serum samples from 115 heart transplant recipients for non-HLA antibodies. The array identified 18 non-HLA antibodies associated with rejection, among which 4 antibodies were not previously described as non-HLA targets. Moreover, the analysis showed that of the 18 identified non-HLA antibodies, 5 predicted rejection and 4 showed a synergistic effect with HLA-DSA. That said, this study did not include pre-transplant serum testing for these non-HLA antibodies, so the absence of these pre-transplant samples makes it difficult to interpret the relation between rejection and the non-HLA antibodies.

Based on the studies discussed above, a clear direction can be observed with regard to the investigation of non-HLA antibodies in heart transplantation. Research currently focuses on utilizing solid-phase assays (i.e., ELISAs and Luminex) for antibody detection, and while these techniques are practical and allow for bulk sample processing with high throughput, they fail to consider donor and organ specificities (**Figure 2**). To our knowledge, only one study described the use of donor-specific human aortic ECs as target cells for a flow cytometry crossmatching assay (68). However, it was unclear whether all of the recipients included in the study were paired with their organ donors for the crossmatching test based on the description of the assay in the methodology section. Moreover, reactivity to IgM AECAs was used as the readout which does not allow for comparisons with other crossmatching tests that report IgG. Therefore, to properly appreciate the role of non-HLA in heart transplantation, more organ- and donor-specific assays are needed.

## Non-HLA Antibodies in Composite Tissue Transplantation

Most hand transplant recipients rarely experience ABMR; however, a few reports have emerged attributing vascular rejection to the presence of anti-AT_1_R and other non-HLA antibodies (47, 48, 69). Banasik et al. investigated the presence of non-HLA antibodies in the post-transplant serum samples of five hand transplant patients (47). The sera were assayed for non-HLA antibodies including AECAs, anti-AT_1_R, and anti-ETAR antibodies. AECAs were detected using the TITERPLANE technique whereby slides coated with HUVECs were incubated with the patient serum, and the reaction of the antigen to IgG, IgA, or IgG was visualized with IIF. Antibodies against AT_1_R and ETAR were detected with commercial ELISAs. Pre-transplant serum samples were also analyzed for the HLA status in all patients using the Luminex technique. Anti-HLA antibodies of class I or II were detected in two patients, albeit these antibodies were not DSA. AECAs were present with moderate activity in only one patient, and both anti-AT1R and anti-ETAR were found with strong reactivity in another patient who had a bilateral transplant and developed six acute rejection episodes. Notably, no association was reported between non-HLA antibodies and HLA, and the repeated acute rejection episodes experienced by the patient with the bilateral transplantation were attributed to the presence of anti-AT1R and anti-ETAR antibodies (47).

Very recently, Sikorska et al. investigated the role of non-HLA antibodies in hand transplant rejection (48). The post-transplant serum samples from six hand transplant recipients were assayed for antibodies against AT_1_R, ETAR, protease-activated receptor 1 (PAR-1), and vascular endothelial growth factor A (VECGF-A) using commercial ELISAs. Additionally, the levels of pro-inflammatory cytokines IL-1, IL-6, and IFNγ were also investigated to evaluate the humoral response post-transplant. The authors reported that repeated episodes of rejection were associated with high levels of anti-AT_1_R and ETAR antibodies as well as increased levels of EC activation markers represented by higher titers of anti-VEGF-A and PAR-1 antibodies in one out of the six included patients. Interestingly, this patient did not develop anti-HLA antibodies. With regard to the pro-inflammatory markers, no elevations were observed for all the three tested cytokines (IL-1, IL-6, IFNγ). Although these findings are promising, it is difficult to highlight the importance of non-HLA in composite tissue transplantation making it difficult to draw firm conclusions at this stage, especially since both of the abovementioned studies base their conclusions on the observation from one single patient.

## Discussion

**Figure 3:**
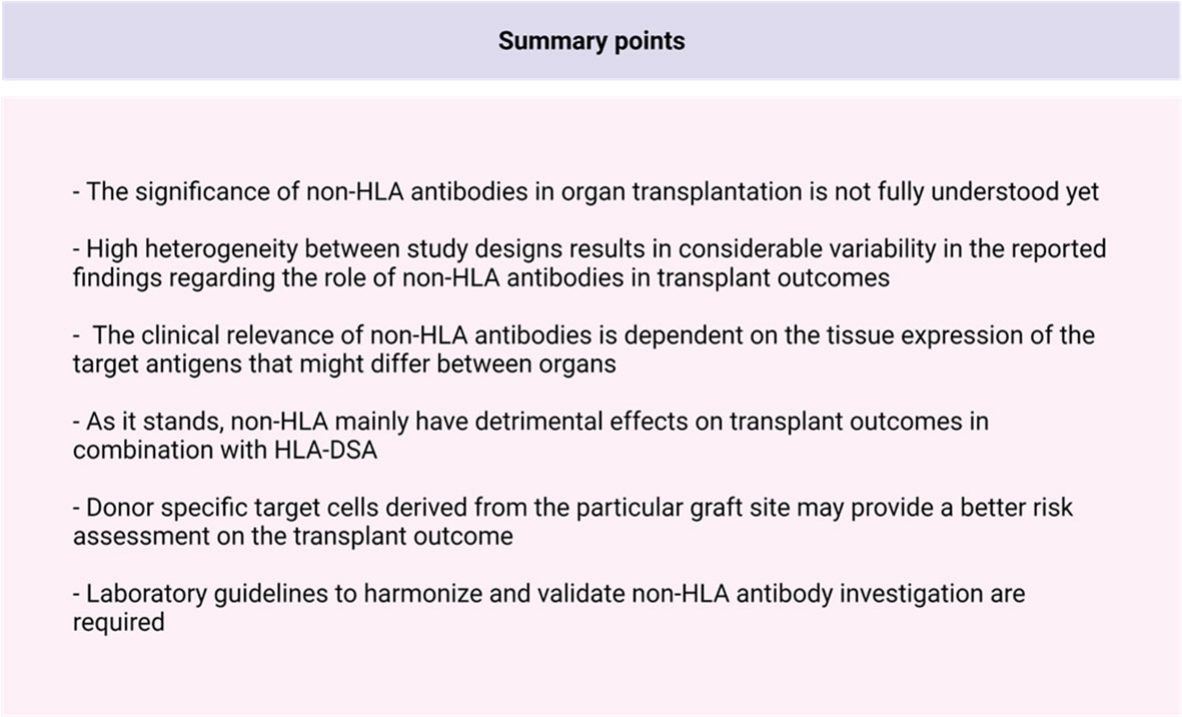
Summary points.

The relevance and importance of non-HLA antibodies in transplantation in addition to HLA-antibodies is increasingly being acknowledged (**Figure 3**). In the past decade, several non-HLA detection and screening assays have been developed, resulting in the identification of multiple non-HLA antibodies. Non-HLA antibodies are associated with a wide range of autoimmune diseases but can also be produced as antibodies after transplantation, possibly due to increased antigen exposure in the context of tissue damage (20, 70–72). It has also been shown that the process of allosensitization to minor histocompatibility non-HLA antigens after previous kidney transplantation affects long-term graft outcomes (36, 73). Most of the potential non-HLA target antigens are ubiquitously expressed throughout the body, resulting in the possible involvement of non-HLA antibodies in all organ transplantations. However, non-HLA antibodies have most extensively been studied in kidney transplantation. Within this line of research, the focus was mostly on the presence of anti-AT_1_R antibodies in the serum of transplant patients and the effect of several non-HLA antibodies on the allogenic endothelium (70, 74). It has been proposed that anti-AT_1_R and anti-HLA antibodies have a synergistic role in mediating kidney allograft rejection through the induction of overexpression of HLA molecules after binding to the ECs (19). AECAs can be detected with flow cytometry, ELISA, indirect immunofluorescence, and high-density protein array as described in this review, and most assays use either EPCs isolated from peripheral blood or HUVECs as an antigen substrate (25, 37, 75, 76). A commercial kit for EC crossmatching is available, and it uses magnetic coated beads against angiopoietin receptor Tie-2 to isolate EPCs (XM-ONE). However, as discussed in the section describing EC-based assays, the current detection methods have several significant limitations. Many of the assays discussed in this review have reported non-HLA antibodies with variable readouts (IgG, IgM, C1q) which might, in turn, make it difficult to compare and correlate the results obtained from different assays and studies. The lack of uniform readouts, cutoffs, and the potential confounders by the presence of HLA antibodies (or other non-HLA antibodies) results in studies describing conflicting relations between non-HLA and rejection and/or graft survival, as described in this review. Moreover, it is yet to be resolved whether non-HLA antibodies mediate graft injury themselves or whether they are produced due to processes following tissue damage. Up until now, it seems that non-HLA mainly have detrimental effects in combination with HLA-DSA. Finally, an ideal antigen identification technique requires a considerable degree of sensitivity and specificity. It must be easily reproducible and can be used to screen samples in bulk (i.e., high throughput). Although the techniques discussed in this report fulfil some of these requirements, an ideal antigen detection technique is yet to be developed.

All in all, the identification of new non-HLA antibodies on endothelial cells and other cell types involved in the transplanted organ and their relevance in transplantation require a rigorous step-by-step scientific process, combining relevant experimental models with clinical investigations in adequately phenotyped, preferably prospective cohorts. The data on the involvement of non-HLA in other solid-organ transplantations than the kidney are scarce and might need more attention. The use of organ donor-derived cells allows for the detection of specific antibody responses against polymorphic proteins that are mismatched between the donor and the recipient. The development of a library of organ-specific endothelial cell lines that are devoid of endogenous HLA expression would be necessary. This organ-specific non-HLA endothelial cell crossmatch assay could eventually help identify different non-HLA risk profiles for rejection and impaired graft survival and should be applied in a systematic fashion in all types of solid-organ transplantations.

In conclusion, several *in-vitro* non-HLA assays have been developed for the screening of non-HLA antibodies pre- and/or post-allograft transplantation, mainly in kidney transplantation. Although some of the published studies have common characteristics between the studied cohorts, there is considerable clinical variability among the patient groups. There are currently barely any laboratory guidelines for the investigation of patients with non-HLA antibodies, resulting in a considerable variability on the accuracy of investigation and heterogeneous study populations that are compared with each other. Additionally, the reported clinical significance for organ transplantation of the non-HLA antibody is variable and is very much dependent on tissue expression of its target antigen, its relationship with HLA-DSA, and the inflammatory context in which it developed. Therefore, not all non-HLA antibodies have pathological relevance. To be able to fully elucidate the clinical relevance of non-HLA antibodies, harmonization and validation of existing non-HLA assays is necessary, in addition to a rigorous step-by-step scientific process to identify and test for new and relevant non-HLA antibodies.

## Author Contributions

RL, DA, and SB designed the manuscript. RL and DA performed the literature search and wrote the manuscript. BH, J-SS, JB, and SB read, corrected, and approved the submitted version.

## Funding

This study was supported by the Dutch Kidney Foundation (grant number: CP1801).

## Conflict of Interest

The authors declare that the research was conducted in the absence of any commercial or financial relationships that could be construed as a potential conflict of interest.

## Publisher’s Note

All claims expressed in this article are solely those of the authors and do not necessarily represent those of their affiliated organizations, or those of the publisher, the editors and the reviewers. Any product that may be evaluated in this article, or claim that may be made by its manufacturer, is not guaranteed or endorsed by the publisher.

## References

[1] Halloran PF Merino Lopez M Barreto Pereira A. Identifying Subphenotypes of Antibody-Mediated Rejection in Kidney Transplants. Am J Transplant (2016) 16:908–20. doi: 10.1111/ajt.13551 26743766

[2] Budding K van de Graaf EA Otten HG. Humoral Immunity and Complement Effector Mechanisms After Lung Transplantation. Transplant Immunol (2014) 31:260–5. doi: 10.1016/j.trim.2014.08.006 25195091

[3] Colvin MM Cook JL Chang P Francis G Hsu DT Kiernan MS. Antibody-Mediated Rejection in Cardiac Transplantation: Emerging Knowledge in Diagnosis and Management: A Scientific Statement From the American Heart Association: Endorsed by the International Society for Heart and Lung Transplantation. Circulation (2015) 131:1608–39. doi: 10.1161/CIR.0000000000000093 25838326

[4] Taner T Stegall MD Heimbach JK. Antibody-Mediated Rejection in Liver Transplantation: Current Controversies and Future Directions. Liver Transplant (2014) 20:514–27. doi: 10.1002/lt.23826 24470340

[5] Philogene MC Jackson AM. Non-HLA Antibodies in Transplantation: When do They Matter? Curr Opin Organ Transplant (2016) 21:427–32. doi: 10.1097/MOT.0000000000000335 27258575

[6] Michielsen LA van Zuilen AD Krebber MM Verhaar MC Otten HG. Clinical Value of Non-HLA Antibodies in Kidney Transplantation: Still an Enigma? Transplant Rev (2016) 30:195–202. doi: 10.1016/j.trre.2016.06.001 27395083

[7] Reindl-Schwaighofer R Heinzel A Gualdoni GA Mesnard L Claas FHJ Oberbauer R. Novel Insights Into Non-HLA Alloimmunity in Kidney Transplantation. Transplant Int (2020) 33:5–17. doi: 10.1111/tri.13546 PMC6972536 31650645

[8] Kamburova EG Gruijters ML Kardol-Hoefnagel T Wisse BW Joosten I Allebes WA. Antibodies Against ARHGDIB are Associated With Long-Term Kidney Graft Loss. Am J Transplant (2019) 19:3335–44. doi: 10.1111/ajt.15493 PMC6899679 31194283

[9] Sánchez-Zapardiel E Castro-Panete MJ Mancebo E Morales P Laguna-Goya R Morales JM. Early Renal Graft Function Deterioration in Recipients With Preformed Anti-MICA Antibodies: Partial Contribution of Complement-Dependent Cytotoxicity. Nephrol Dialysis Transplant (2016) 31:150–60. doi: 10.1093/ndt/gfv308 26323481

[10] Clotet-Freixas S Kotlyar M McEvoy CM Pastrello C Rodríguez-Ramírez S Farkona S. Increased Autoantibodies Against Ro/SS-A, CENP-B, and La/SS-B in Patients With Kidney Allograft Antibody-Mediated Rejection. Transplant Direct (2021) 7:e768–8. doi: 10.1097/TXD.0000000000001215 PMC8454907 34557585

[11] Pearl MH Chen L ElChaki R Elashoff D Gjertson DW Rossetti M. Endothelin Type A Receptor Antibodies Are Associated With Angiotensin II Type 1 Receptor Antibodies, Vascular Inflammation, and Decline in Renal Function in Pediatric Kidney Transplantation. Kidney Int Rep (2020) 5:1925–36. doi: 10.1016/j.ekir.2020.09.004 PMC7609952 33163713

[12] Philogene MC Bagnasco S Kraus ES Montgomery RA Dragun D Leffell MS. Anti-Angiotensin II Type 1 Receptor and Anti-Endothelial Cell Antibodies: A Cross-Sectional Analysis of Pathological Findings in Allograft Biopsies. Transplantation (2017) 101:608–15. doi: 10.1097/TP.0000000000001231 PMC5319389 27222934

[13] Jurcevic S Ainsworth ME Pomerance A Smith JD Robinson DR Dunn MJ. Antivimentin Antibodies Are an Independent Predictor of Transplant-Associated Coronary Artery Disease After Cardiac Transplantation1. Transplantation (2001) 71:886–92. doi: 10.1097/00007890-200104150-00011 11349721

[14] Xu Q McAlister VC House AA Molinari M Leckie S Zeevi A. Autoantibodies to LG3 are Associated With Poor Long-Term Survival After Liver Retransplantation. Clin Transplant (2021) 35:e14318. doi: 10.1111/ctr.14318 33871888

[15] Philogene MC Zhou S Lonze BE Bagnasco S Alasfar S Montgomery RA. Pre-Transplant Screening for Non-HLA Antibodies: Who Should be Tested? Hum Immunol (2018) 79:195–202. doi: 10.1016/j.humimm.2018.02.001 29428484

[16] Zhang Q Reed EF. The Importance of non-HLA Antibodies in Transplantation. Nat Rev Nephrol (2016) 12:484–95. doi: 10.1038/nrneph.2016.88 PMC5669045 27345243

[17] Cuevas E Arreola-Guerra JM Hernández-Méndez EA Salcedo I Castelán N Uribe-Uribe NO. Pretransplant Angiotensin II Type 1-Receptor Antibodies are a Risk Factor for Earlier Detection of *De Novo* HLA Donor-Specific Antibodies. Nephrol Dialysis Transplant (2016) 31:1738–45. doi: 10.1093/ndt/gfw204 27220757

[18] Gareau AJ Wiebe C Pochinco D Gibson IW Ho J Rush DN. Pre-Transplant AT1R Antibodies Correlate With Early Allograft Rejection. Transplant Immunol (2018) 46:29–35. doi: 10.1016/j.trim.2017.12.001 29217423

[19] Dragun D Catar R Philippe A. Non-HLA Antibodies Against Endothelial Targets Bridging Allo- and Autoimmunity. Kidney Int (2016) 90:280–8. doi: 10.1016/j.kint.2016.03.019 27188505

[20] Cardinal H Dieudé M Hébert MJ. The Emerging Importance of Non-HLA Autoantibodies in Kidney Transplant Complications. J Am Soc Nephrol (2017) 28:400–6. doi: 10.1681/ASN.2016070756 PMC5280028 27798244

[21] Breimer ME Rydberg L Jackson AM Lucas DP Zachary AA Melancon JK. Multicenter Evaluation of a Novel Endothelial Cell Crossmatch Test in Kidney Transplantation. Transplantation (2009) 87:549–56. doi: 10.1097/TP.0b013e3181949d4e 19307793

[22] Senev A Otten HG Kamburova EG Callemeyn J Lerut E Van Sandt V. Antibodies Against ARHGDIB and ARHGDIB Gene Expression Associate With Kidney Allograft Outcome. Transplantation (2020) 104:1462–71. doi: 10.1097/TP.0000000000003005 31651716

[23] Chan AP Guerra MR Rossetti M Hickey MJ Venick RS Marcus EA. Non-HLA AT1R Antibodies are Highly Prevalent After Pediatric Intestinal Transplantation. Pediatr Transplant (2021) 25:e13987. doi: 10.1111/petr.13987 33590644 PMC8058288

[24] Alheim M Johansson SM Hauzenberger D Grufman P Holgersson J. A Flow Cytometric Crossmatch Test for Simultaneous Detection of Antibodies Against Donor Lymphocytes and Endothelial Precursor Cells. Tissue Antigens (2010) 75:269–77. doi: 10.1111/j.1399-0039.2009.01439.x 20070600

[25] Xavier P Aires P Sampaio S Mendes C Monteiro M Alves H. XM-ONE Detection of Endothelium Cell Antibodies Identifies a Subgroup of HLA-Antibody Negative Patients Undergoing Acute Rejection. Transplant Proc (2011) 43:91–4. doi: 10.1016/j.transproceed.2010.12.040 21335162

[26] Soyöz M Kilicaslan Ayna T Çerçi B Özkızılcık Koçyiğit A Pirim I. Comparison of HLA and Non-HLA Antibodies Regarding to Rejection Pattern of the Kidney Transplantation. J Tepecik Educ Res Hosp (2020) 30:156–63. doi: 10.5222/terh.2020.27146

[27] Zitzner JR Shah S Jie C Wegner W Tambur AR Friedewald JJ. A Prospective Study Evaluating the Role of Donor-Specific Anti-Endothelial Crossmatch (XM-ONE Assay) in Predicting Living Donor Kidney Transplant Outcome. Hum Immunol (2013) 74:1431–6. doi: 10.1016/j.humimm.2013.06.007 23777928

[28] Magro CM Deng A Pope-Harman A Waldman WJ Bernard Collins A Adams PW. Humorally Mediated Posttransplantation Septal Capillary Injury Syndrome as a Common Form of Pulmonary Allograft Rejection: A Hypothesis. Transplantation (2002) 74:1273–80. doi: 10.1097/00007890-200211150-00013 12451265

[29] Reinsmoen NL Mirocha J Ensor CR Marrari M Chaux G Levine DJ. A 3-Center Study Reveals New Insights Into the Impact of Non-HLA Antibodies on Lung Transplantation Outcome. Transplantation (2017) 101:1215–21. doi: 10.1097/TP.0000000000001389 27973391

[30] Otten HG van den Bosch JMM van Ginkel WGJ van Loon M van de Graaf EA. Identification of Non-HLA Target Antigens Recognized After Lung Transplantation. J Heart Lung Transplant (2006) 25:1425–30. doi: 10.1016/j.healun.2006.09.022 17178336

[31] Yu S Huh HJ Lee KW Park JB Kim SJ Huh W. Pre-Transplant Angiotensin II Type 1 Receptor Antibodies and Anti-Endothelial Cell Antibodies Predict Graft Function and Allograft Rejection in a Low-Risk Kidney Transplantation Setting. Ann Lab Med (2020) 40:398–408. doi: 10.3343/alm.2020.40.5.398 32311853 PMC7169631

[32] Pontes LFS Carvalho L Stumbo AC Porto LC. Detection and Localization of non-HLA-ABC Antigenic Sites Relevant to Kidney Rejection on Endothelial Cells. J Immunol Methods (2001) 251:73–80. doi: 10.1016/S0022-1759(01)00309-X 11292483

[33] Ming Y Hu J Luo Q Ding X Luo W Zhuang Q. Acute Antibody-Mediated Rejection in Presence of MICA-DSA and Successful Renal Re-Transplant With Negative-MICA Virtual Crossmatch. PloS One (2015) 10:e0127861–. doi: 10.1371/journal.pone.0127861 26024219 PMC4449040

[34] Crespo M Llinàs-Mallol L Redondo-Pachón D Butler C Gimeno J Pérez-Sáez MJ. Non-HLA Antibodies and Epitope Mismatches in Kidney Transplant Recipients With Histological Antibody-Mediated Rejection. Front Immunol (2021) 12:2606. doi: 10.3389/fimmu.2021.703457 PMC8300190 34305943

[35] Sorohan BM Sinescu I Tacu D Bucșa C Țincu C Obrișcă B. Immunosuppression as a Risk Factor for *De Novo* Angiotensin II Type Receptor Antibodies Development After Kidney Transplantation. J Clin Med (2021) 10:5390. doi: 10.3390/jcm10225390 34830672 PMC8625545

[36] Reindl-Schwaighofer R Heinzel A Kainz A van Setten J Jelencsics K Hu K. Contribution of Non-HLA Incompatibility Between Donor and Recipient to Kidney Allograft Survival: Genome-Wide Analysis in a Prospective Cohort. Lancet (2019) 393:910–7. doi: 10.1016/S0140-6736(18)32473-5 30773281

[37] Jackson AM Sigdel TK Delville M Hsieh SC Dai H Bagnasco S. Endothelial Cell Antibodies Associated With Novel Targets and Increased Rejection. J Am Soc Nephrol (2015) 26:1161–71. doi: 10.1681/ASN.2013121277 PMC4413753 25381426

[38] Li L Wadia P Chen R Kambham N Naesens M Sigdel TK. Identifying Compartment-Specific non-HLA Targets After Renal Transplantation by Integrating Transcriptome and “Antibodyome” Measures. Proc Natl Acad Sci United States America (2009) 106:4148–53. doi: 10.1073/pnas.0900563106 PMC2657434 19251643

[39] Kamburova EG Kardol-Hoefnagel T Wisse BW Joosten I Allebes WA van der Meer A. Development and Validation of a Multiplex Non-HLA Antibody Assay for the Screening of Kidney Transplant Recipients. Front Immunol (2018) 9. doi: 10.3389/fimmu.2018.03002 PMC6315148 30631326

[40] Lamarthée B Kardol-Hoefnagel T Wisse BW Joosten I Allebes WA van der Meer A. CRISPR/Cas9-Engineered HLA-Deleted Glomerular Endothelial Cells as a Tool to Predict Pathogenic Non-HLA Antibodies in Kidney Transplant Recipients. J Am Soc Nephrol (2021) 32:3231. doi: 10.1681/ASN.2021050689 35167486 PMC8638404

[41] Ekong UD Antala S Bow UD Sese D Morotti R Rodriguez-Davalos M. HLA, non-HLA Antibodies, and Eplet Mismatches in Pediatric Liver Transplantation: Observations From a Small, Single-Center Cohort. Exp Clin Transplant (2019) 17:6–17. doi: 10.6002/ect.MESOT2018.L30 PMC11165937 30777518

[42] Ohe H Uchida Y Yoshizawa A Hirao H Taniguchi M Maruya E. Association of Anti-Human Leukocyte Antigen and Anti-Angiotensin II Type 1 Receptor Antibodies With Liver Allograft Fibrosis After Immunosuppression Withdrawal. Transplantation (2014) 98:1105–11. doi: 10.1097/TP.0000000000000185 24914568

[43] O’Leary JG Demetris AJ Philippe A Freeman R Cai J Heidecke H. Non-HLA Antibodies Impact on C4d Staining, Stellate Cell Activation and Fibrosis in Liver Allografts. Transplantation (2017) 101:2399–409. doi: 10.1097/TP.0000000000001853 28665894

[44] Hiemann NE Meyer R Wellnhofer E Schoenemann C Heidecke H Lachmann N. Non-HLA Antibodies Targeting Vascular Receptors Enhance Alloimmune Response and Microvasculopathy After Heart Transplantation. Transplantation (2012) 94:919–24. doi: 10.1097/TP.0b013e3182692ad2 23034559

[45] Zhang Q Cecka JM Gjertson GW Ge P Rose ML Patel JK. HLA and MICA: Targets of Antibody-Mediated Rejection in Heart Transplantation. Transplantation (2011) 91:1153–8. doi: 10.1097/TP.0b013e3182157d60 PMC3563270 21544036

[46] Butler CL Hickey MJ Jiang N Zheng Y Gjertson D Zhang Q. Discovery of Non-HLA Antibodies Associated With Cardiac Allograft Rejection and Development and Validation of a non-HLA Antigen Multiplex Panel: From Bench to Bedside. Am J Transplant (2020) 20:2768–80. doi: 10.1111/ajt.15863 PMC7494540 32185871

[47] Banasik M Jabłecki J Boratyńska M Kamińska D Kościelska-Kasprzak K Bartoszek D. Humoral Immunity in Hand Transplantation: Anti-HLA and Non-HLA Response. Hum Immunol (2014) 75:859–62. doi: 10.1016/j.humimm.2014.06.010 24952209

[48] Sikorska D Kamińska D Catar R Banasik M Heidecke H Schulze-Forster K. Non-HLA Antibodies in Hand Transplant Recipients Are Connected to Multiple Acute Rejection Episodes and Endothelial Activation. J Clin Med (2022) 11:833. doi: 10.3390/jcm11030833 35160284 PMC8837026

[49] Moraes JR Pettaway C Stastny P. Prediction of Early Kidney Transplant Rejection By a Crossmatch With Donor Skin. Transplantation (1989) 48:951–2. doi: 10.1097/00007890-198912000-00010 2688207

[50] Vermehren D Sumitran-Holgersson S. Isolation of Precursor Endothelial Cells From Peripheral Blood for Donor-Specific Crossmatching Before Organ Transplantation. Transplantation (2002) 74:1479–86. doi: 10.1097/00007890-200212150-00001 12490778

[51] Sumitran-Karuppan S Tyden G Reinholt F Berg U Moller E. Hyperacute Rejections of Two Consecutive Renal Allografts and Early Loss of the Third Transplant Caused by non-HLA Antibodies Specific for Endothelial Cells. Transplant Immunol (1997) 5:321–7. doi: 10.1016/S0966-3274(97)80016-0 9504155

[52] Lammerts RGM Lagendijk LM Tiller G Dam WA Lancaster HL Daha MR. Machine-Perfused Donor Kidneys as a Source of Human Renal Endothelial Cells. Am J Physiology-Renal Physiol (2021) 320:F947–62. doi: 10.1152/ajprenal.00541.2020 33719571

[53] Delville M Lamarthée B Pagie S See SB Rabant M Burger C. Early Acute Microvascular Kidney Transplant Rejection in the Absence of Anti-HLA Antibodies Is Associated With Preformed IgG Antibodies Against Diverse Glomerular Endothelial Cell Antigens. J Am Soc Nephrology : JASN (2019) 30:692–709. doi: 10.1681/ASN.2018080868 PMC6442343 30850439

[54] Aird WC. Endothelium in Health and Disease. Pharmacol Rep (2008) 60:139–43. 18276995

[55] Molema G Zijlstra JG van Meurs M Kamps JAAM. Renal Microvascular Endothelial Cell Responses in Sepsis-Induced Acute Kidney Injury. Nat Rev Nephrol (2021) 18:95–112. doi: 10.1038/s41581-021-00489-1 34667283

[56] Gunawardana H Romero T Yao N Heidt S Mulder A Elashoff DA. Tissue-Specific Endothelial Cell Heterogeneity Contributes to Unequal Inflammatory Responses. Sci Rep (2021) 11:1–20. doi: 10.1038/s41598-020-80102-w 33479269 PMC7820348

[57] Shoenfeld Y. Classification of Anti-Endothelial Cell Antibodies Into Antibodies Against Microvascular and Macrovascular Endothelial Cells: The Pathogenic and Diagnostic Implications. Cleveland Clinic J Med (2002) 69:1484–94. doi: 10.3949/ccjm.69.Suppl_2.SII65 12086268

[58] Lion J Taflin C Cross AR Robledo-Sarmiento M Mariotto E Savenay A. HLA Class II Antibody Activation of Endothelial Cells Promotes Th17 and Disrupts Regulatory T Lymphocyte Expansion. Am J Transplant (2016) 16:1408–20. doi: 10.1111/ajt.13644 26614587

[59] Valenzuela NM Mulder A Reed EF. HLA Class I Antibodies Trigger Increased Adherence of Monocytes to Endothelial Cells by Eliciting an Increase in Endothelial P-Selectin and, Depending on Subclass, by Engaging Fcγrs. J Immunol (2013) 190:6635–50. doi: 10.4049/jimmunol.1201434 PMC3885237 23690477

[60] Leisman DE Fernandes TD Bijol V Abraham MN Lehman JR Taylor MD. Impaired Angiotensin II Type 1 Receptor Signaling Contributes to Sepsis-Induced Acute Kidney Injury. Kidney Int (2021) 99:148–60. doi: 10.1016/j.kint.2020.07.047 PMC8030124 32882263

[61] Dragun D. The Detection of Antibodies to the Angiotensin II-Type 1 Receptor in Transplantation. In: Transplantation Immunology. Berlin, Germany:Springer (2013). p. 331–3. 10.1007/978-1-62703-493-7_19 23775747

[62] Reinsmoen NL Lai CH Heidecke H Haas M Cao K Ong G. Anti-Angiotensin Type 1 Receptor Antibodies Associated With Antibody Mediated Rejection in Donor HLA Antibody Negative Patients. Transplantation (2010) 90:1473–7. doi: 10.1097/TP.0b013e3181fd97f1 21030904

[63] Sboner A Karpikov A Chen G Smith M Mattoon D Freeman-Cook L. Robust-Linear-Model Normalization To Reduce Technical Variability in Functional Protein Microarrays. J Proteome Res (2009) 8:5451–64. doi: 10.1021/pr900412k 19817483

[64] Terasaki PI Ozawa M Castro R. Four-Year Follow-Up of a Prospective Trial of HLA and MICA Antibodies on Kidney Graft Survival. Am J Transplant (2007) 7:408–15. doi: 10.1111/j.1600-6143.2006.01644.x 17229080

[65] Zou Y Stastny P Süsal C Döhler B Opelz G. Antibodies Against MICA Antigens and Kidney-Transplant Rejection. New Engl J Med (2007) 357:1293–300. doi: 10.1056/NEJMoa067160 17898098

[66] Zhou S Yi T Zhang B Huang F Huang H Tang J. Mapping the High Throughput SEREX Technology Screening for Novel Tumor Antigens. Combinatorial Chem High Throughput Screening (2012) 15:202–15. doi: 10.2174/138620712799218572 22221053

[67] Zhang X Levine R Patel JK Kittleson M Czer L Kobashigawa JA. Association of Vimentin Antibody and Other Non-HLA Antibodies With Treated Antibody Mediated Rejection in Heart Transplant Recipients. Hum Immunol (2020) 81:671–4. doi: 10.1016/j.humimm.2020.09.003 33041085

[68] Hosenpud JD Mauck KA Hogan KB. Cardiac Allograft Vasculopathy: IgM Antibody Responses to Donor-Specific Vascular Endothelium: 1. Transplantation (1997) 63:1602–6. doi: 10.1097/00007890-199706150-00011 9197353

[69] Dwyer KM Carroll R Hill P Bateman S Baker C Langham RG. Refractory Vascular Rejection in a Hand Allograft in the Presence of Antibodies Against Angiotensin II (Type 1) Receptor. Transplantation (2017) 101:e344–5. doi: 10.1097/TP.0000000000001904 28767535

[70] Dragun D Müller DN Bräsen JH Fritsche L Nieminen-Kelhä M Dechend R. Angiotensin II Type 1–Receptor Activating Antibodies in Renal-Allograft Rejection. New Engl J Med (2005) 352:558–69. doi: 10.1056/NEJMoa035717 15703421

[71] Mahesh B Leong H-S McCormack A Sarathchandra P Holder A Rose ML. Autoantibodies to Vimentin Cause Accelerated Rejection of Cardiac Allografts. Am J Pathol (2007) 170:1415–27. 10.2353/ajpath.2007.060728 PMC1829474 17392180

[72] Magro CM Ross P Marsh CB Allen JN Liff D Knight DA. The Role of Anti-Endothelial Cell Antibody-Mediated Microvascular Injury in the Evolution of Pulmonary Fibrosis in the Setting of Collagen Vascular Disease. Am J Clin Pathol (2007) 127:237–47. doi: 10.1309/CNQDMHLH2WGKL32T 17210529

[73] Steers NJ Li Y Drace Z D’Addario JA Fischman C Liu L. Genomic Mismatch at LIMS1 Locus and Kidney Allograft Rejection. New Engl J Med (2019) 380:1918–28. doi: 10.1056/NEJMoa1803731 PMC6589355 31091373

[74] Lefaucheur C Louis K Philippe A Loupy A Coates PT. The Emerging Field of Non–Human Leukocyte Antigen Antibodies in Transplant Medicine and Beyond. Kidney Int (2021) 100:787–98. doi: 10.1016/j.kint.2021.04.044 34186057

[75] Sun Q Cheng Z Cheng D Chen J Ji S Wen J. *De Novo* Development of Circulating Anti-Endothelial Cell Antibodies Rather Than Pre-Existing Antibodies Is Associated With Post-Transplant Allograft Rejection. Kidney Int (2011) 79:655–62. doi: 10.1038/ki.2010.437 20980975

[76] Han F Lv R Jin J Chen Y Wang H Chen J. Pre-Transplant Serum Concentrations of Anti-Endothelial Cell Antibody in Panel Reactive Antibody Negative Renal Recipients and its Impact on Acute Rejection. Clin Chem Lab Med (2009) 47:1265–9. doi: 10.1515/CCLM.2009.283 19751142

